# Intrauterine hematoma evacuation during fetoscopic myelomeningocele repair: a novel application of the NICO Myriad

**DOI:** 10.3171/2026.1.FOCVID25233

**Published:** 2026-04-01

**Authors:** Raj Swaroop Lavadi, Michael C. Sundahl, Kyle Ortiz Rodriguez, Brittany J. Arkerson, Anas Aljabari, Laurie L. Ackerman, Rabia Qaiser, Jignesh Tailor, Thomas Larrew, Charles B. Stevenson, Ramen H. Chmait, Hiba J. Mustafa, Jason Chu

**Affiliations:** 1 Department of Neurological Surgery, Indiana University School of Medicine, Indianapolis;; 2 The Fetal Center at Indiana University and Riley Children’s Hospital, Division of Maternal-Fetal Medicine, Department of Obstetrics and Gynecology, Indiana University School of Medicine, Indianapolis, Indiana;; 3 Division of Maternal-Fetal Medicine, Department of Obstetrics and Gynecology, Keck School of Medicine, University of Southern California, Los Angeles, California; and; 4 Division of Pediatric Neurosurgery, Riley Children’s Hospital, Indiana University School of Medicine, Indianapolis, Indiana

**Keywords:** spina bifida, myelomeningocele, repair, fetal surgery, NICO Myriad, hemostasis

## Abstract

Evacuating intrauterine hematomas during fetoscopic myelomeningocele repair can be challenging. The NICO Myriad (Stryker Inc.) has been previously described to resect brain tumors, as an adjunct in intraventricular neuroendoscopy, and, most recently, has been proven to effectively remove intracerebral hematomas at clinical and cost-effective significance. This device has not been described in fetal neurosurgery. In this video, the NICO Myriad was utilized during a minilaparotomy fetal myelomeningocele repair to mechanically aspirate an intrauterine hematoma noted at the end of the operation. The surgical technique and technical nuances of using the NICO Myriad during fetoscopic myelomeningocele repair are reviewed.

The video can be found here: https://stream.cadmore.media/r10.3171/2026.1.FOCVID25233

## Transcript

### 0:20

In this video, we demonstrate our experience evacuating an intrauterine hematoma encountered during a fetoscopic myelomeningocele repair using the NICO Myriad.

### 0:31

Fetal surgery for myelomeningocele has increased in popularity since the publication of the Management of Myelomeningocele trial in 2011.^1^ Over the last decade, there has been an evolution of surgical technique, from the classic open hysterotomy for fetal myelomeningocele repair to a fetoscopic approach.

### 0:49

The percutaneous or minilaparotomy fetoscopic repair technique was described by Chmait et al.^2^ in 2022. Note that the uterus is maintained within the abdomen and not exteriorized as described in other fetoscopic techniques. Advantages of this minimally invasive technique include a smaller incision, less maternal pain, and a shorter hospital course. At Indiana University and the Riley Hospital for Children, we have adopted this technique with slight variation to accommodate our laparoscope and our laparoscopic instruments.

### 1:21

The minilaparotomy is completed through a 3- to 4-cm incision and dissection, carried down through the anterior abdominal wall to the peritoneum. An Alexis-XS retractor is placed to maintain visualization of the uterus. Several sutures are placed for uterine membrane plication in a box fashion. An 18-gauge needle is placed intrauterine, and the Seldinger technique is used to place a guidewire, followed by a 12-French dilator and a laparoscopic port.

### 1:48

Two additional 11-French ports are placed percutaneously with ultrasound guidance into the uterus. Ultrasound visualization of the insertion is critical to visualize and avoid the uterine vasculature on entry, ensure appropriate depth and orientation of the ports, as well as to avoid placental injury. These ports are secured to the skin. Here is a picture after placement of all three ports for fetoscopic repair.

### 2:11

After release of the neural placode, we complete a three-layer closure. The first layer is a duraplasty onlay to cover the placode and reconstruct the thecal sac. The second layer is a myofascial flap, closed in a running fashion to provide an additional layer of coverage over the myelomeningocele repair site. Finally, the skin is closed with running sutures. Our group utilizes Quill, a barbed suture that does not require intrauterine or extracorporeal knot-tying. Once complete, all uterine entry sites are closed and a standard maternal abdominal closure is conducted. The picture in the bottom right shows the final maternal incision minilaparotomy and the two percutaneous incisions.

### 2:55

As groups transition to the fetoscopic technique for MMC repair, several limitations arise. First, the degree of surgical freedom is restricted to the linear visualization of the laparoscope. Second, there is a learning curve as groups transition from the classic open hysterotomy approach to fetoscopic repair. New surgical skills must also be acquired for laparoscopic dissection and suturing. Finally, there are limited options for intrauterine hemostasis control and intrauterine hematoma removal during fetoscopic cases.^3^ The presence of a postoperative intrauterine hematoma increases the risk of preterm, premature rupture of membranes. In this case, we demonstrate the use of the NICO Myriad during fetoscopic MMC repair for intrauterine hematoma evacuation noted at the end of the surgery.

### 3:44

The NICO Myriad is a minimally invasive suction aspiration system. It uses nonablative mechanical cutting with user-controlled variable aspiration.^4^ The device also has directionality that allows the aspiration mouth to rotate without turning the instrument. It offers two modes: a suction-aspiration mode and a suction-only mode. The Myriad is a commonly used instrument in neurosurgery.^5,6^ It has not been described in fetal neurosurgery.

### 4:11

This is a case of a 22-year-old female that underwent fetoscopic myelomeningocele repair at 25 weeks of gestational age. The fetus had a large T10–S4 myeloschisis type of lesion. Prenatal ultrasound demonstrated an S1 motor level with good movement in the legs, including dorsi- and plantar flexion. The fetus was also noted to have significant ventriculomegaly with a grade III hindbrain herniation. Prenatal counseling of fetal myelomeningocele repair included preservation of lower extremity neurological function, potential for hindbrain herniation reversal, and an increased risk for hydrocephalus with ventriculomegaly > 15 mm. The fetus underwent a fetoscopic MMC repair via the minilaparotomy technique with a successful three-layer closure.

### 5:01

After the repair was completed and during final irrigation, we noted two large intrauterine hematomas. Of note, we believe the hematoma was maternal in origin and not a result of blood loss from the fetus. The most likely source was uterine bleeding during placement of one of the laparoscopic ports, and this was controlled immediately with uterine myometrial sutures.

### 5:23

The suction irrigator was initially used to break up these clots and to aspirate them. This was aided by a second instrument to assist with clot morselization. Although there was a reduction in clot burden, some of these were too large to be removed with either the suction or through the laparoscopic ports.

### 5:40

The remaining hematoma was then consolidated along the caudal aspect of the fetus. The lower uterine quadrants were inspected and irrigated to identify any remaining hematoma.

### 5:51

We introduced the NICO Myriad through one of the 11-French ports. The aspirating mouth can be rotated with a handpiece and directed towards the hematoma. The NICO Myriad can then be used to mechanically aspirate the remainder of the hematoma. In the suction-aspiration mode, there is gentle, user-controlled suction that can deliver the hematoma to the aspirating mouth. The gelatinous consistency of the hematoma is ideal for mechanical aspiration with the NICO Myriad. In this close-up view, the mechanical aspirating mouth of the NICO Myriad is seen in action. Visualization of the direction of the mouth is of paramount importance to avoid accidental injury to the adjacent structures during the case. In the instance of intrauterine fetal myelomeningocele surgery, the integrity of the uterine membranes, placenta, umbilical cord, and the fetus must be preserved. The NICO Myriad also has a suction-only feature without mechanical aspiration. This can be utilized to deliver the hematoma away from the uterine membrane to a more accommodating location. Remnant clot that may be unsafe to aspirate can also be mobilized with the assistance of a second laparoscopic instrument. The NICO Myriad can be reintroduced to remove the clot. Note that the aspiration mouth is visualized in the "up" orientation, with the instrument resting on the fetus, to avoid inadvertent injury to the back and surgical repair site. Bimanual technique can also be used to mobilize any remaining hematoma that can later be aspirated.

### 7:15

Once hematoma removal is complete, the intrauterine cavity and fetus are irrigated with warm lactated Ringer’s. The prior uterine quadrants were reinspected, noted to be free of hematoma, and the irrigation returned clear fluid.

### 7:29

Postoperatively, the patient had done well without any immediate complications. They were mobilizing on postoperative day 1 with minimal pain. They met discharge criteria from the Maternal-Fetal Medicine service on postoperative day 3. Unfortunately, the patient returned approximately 6 weeks later with premature rupture of membranes, and the infant was delivered at 31 weeks via a normal spontaneous vaginal delivery. At the time of delivery, the back was noted to be completely healed without evidence of CSF leak. On neurological exam, they were noted to have S1 motor function despite a T10 anatomical level of the lesion. Postoperative MRI demonstrated ventriculomegaly and complete hindbrain herniation reversal. They were admitted to the neonatal intensive care unit for approximately 8 weeks, but did not develop symptoms of hydrocephalus. At 4 months of age, the infant continues to do well with a well-healed incision and without having met the revised Tulipan criteria for CSF diversion.^7^

### 8:29

Fetal surgery for myelomeningocele has increased in popularity since the publication of the MOMs trial, with a recent trend toward fetoscopic repair. Intrauterine hematoma evacuation may be limited during fetoscopic myelomeningocele surgery. The NICO Myriad is a mechanical aspiration device commonly used during neurosurgical procedures and can be adapted for use in fetoscopic myelomeningocele surgery. Care must be used to avoid injury to the uterine membranes. The suction on the NICO Myriad can mobilize clots prior to mechanical aspiration. Bimanual technique with an additional laparoscopic instrument assists with safety while using the NICO Myriad.

### 9:08

Here are the references that have been used to guide this video publication.

## Disclosures

The authors report no conflict of interest concerning the materials or methods used in this study or the findings specified in this publication.

## Author Contributions

Primary surgeon: Mustafa, Chu. Assistant surgeon: Arkerson. Editing and drafting the video and abstract: Lavadi, Sundahl, Ortiz Rodriguez, Aljabari, Stevenson, Chu. Critically revising the work: Lavadi, Sundahl, Ortiz Rodriguez, Qaiser, Larrew, Stevenson, Chmait. Reviewed submitted version of the work: Lavadi, Sundahl, Ortiz Rodriguez, Arkerson, Ackerman, Qaiser, Tailor, Larrew, Stevenson, Chmait, Chu. Approved the final version of the work on behalf of all authors: Lavadi. Supervision: Chu.

## Supplemental Information

### Patient Informed Consent

The necessary patient informed consent was obtained in this study.

## References

[1] Adzick NS Thom EA Spong CY A randomized trial of prenatal versus postnatal repair of myelomeningocele N Engl J Med 2011 364 11 993 1004 10.1056/NEJMoa1014379 21306277 PMC3770179

[2] Chmait RH Monson MA Pham HQ Percutaneous/mini-laparotomy fetoscopic repair of open spina bifida: a novel surgical technique Am J Obstet Gynecol 2022 227 3 375 383 10.1016/j.ajog.2022.05.032 35752302

[3] Stevenson CB Fletcher S Larrew T Chu JK In-utero repair of open neural tube defects, lesion closure techniques and the choice of patch Best Pract Res Clin Obstet Gynaecol 2025 103 102677 10.1016/j.bpobgyn.2025.102677 41240491

[4] Nico technologies. Stryker Accessed February 19, 2026 https://www.stryker.com/us/en/nse/campaigns/nico-technologies.html

[5] Dlouhy BJ Dahdaleh NS Greenlee JD Emerging technology in intracranial neuroendoscopy: application of the NICO Myriad Neurosurg Focus 2011 30 4 E6 10.3171/2011.2.FOCUS10312 21456933

[6] Pradilla G Ratcliff JJ Hall AJ Trial of early minimally invasive removal of intracerebral hemorrhage N Engl J Med 2024 390 14 1277 1289 10.1056/NEJMoa2308440 38598795

[7] Tulipan N Wellons JC III Thom EA Prenatal surgery for myelomeningocele and the need for cerebrospinal fluid shunt placement J Neurosurg Pediatr 2015 16 6 613 620 10.3171/2015.7.PEDS15336 26369371 PMC5206797

